# Editorial Notes, News and Miscellany

**Published:** 1912-07

**Authors:** 


### Editorial Notes, News and Miscellany.

Fee Splitting Tabooed in Texas.—The State Medical Association has made fee splitting "an offense punishable by reprimand, suspension or expulsion" of a member.

"Not in Politics."—The *Journal* of the State Medical Association says: "Neither the *Journal* nor the Association is in politics," and then proceeds to rap Colquitt and boom Ramsey and Looney.

Dr. R. H. L. Bibb, our associate editor, with his family, will spend the summer at their old home at Saltillo, Mexico, to escape the heat of Austin. Me,—I'm a sorter Salamander, and couldn't get away, if I wasn't.

Dr. Herff was the first in the United States to trephine for epilepsy. He performed the first lithotomy in Texas. He did the first cataract operation in Texas. He performed the first hysterectomy and the first gastrotomy in the United States. He performed his last surgical operation at 87 years of age. (See June number.)

From Atlantic City.—Dr. J. A. Witherspoon, of Nashville, was elected President A. M. A. Dr. Thos. Stedman of the *New York Medical Record* was elected President of the Medical Editors' Association. Macdonald re-elected Secretary and Treasurer, and Dr. H. E. Lewis, of *American Medicine,* was elected to edit a journal which the Association will issue bimonthly, in place of a bound volume annually, as heretofore.

The Mosquito Delenda Est(?)—Dr. C. A. Campbell, of San Antonio, would pass a law to protect the leather-wing bat, "the natural enemy of the mosquito." Why not *prevent* the mosquito? The English have exterminated him the entire length of the Blue Nile in Africa, why not we, in Texas? The bat is a breeder of mites, which make life a burden in our public buildings, and it would be a blessing if we could find *his* natural enemy and compass his extinction.

THE SCAPEGOAT.—Teacher—"I shall not keep you after school, Johnnie. You may go home now."

Johnnie—"I don't want ter go home. There's a baby just come to our house."

Teacher—"You ought to be glad, Johnnie. A dear little baby ——"

Johnnie (vehemently)—"I ain't glad. Pa'll blame me—he blames me for everything."—*Lippincott's Magazine.*

---

A PUBLICATION that has held its subscription list and its largest and best advertisers continuously *more than a quarter century* as has the "Red Back," despite the free State medical journal and the numerous free bulletins ought to be—and is—the *best advertising medium for doctors in Texas.* The advertisements are read, and the journals are filed and preserved for reference. Drummers tell me they see the "Red Back" everywhere, open, while scores of other journals are piled up in a corner, with their wrappers on. The doctors read everything in the "Red Back," ads. and all. Try it a year.

---

I LIKE YOUR independent and original attitude in medical journalism. I have read your special articles with interest. I am heartily in accord with the idea of sterilizing the hopelessly incompetent and unfit, and have written a number of times on this subject for different publications.

DR. W. T. MARRS, Peoria, Ill.

---

LOUISVILLE, KY., June 4, 1912.

*Dr. F. E. Daniel, Austin, Texas.*

DEAR SIR: We thank you very much for your kindness and liberality, and hope that we may both live many years to enjoy the fruits of our mutual labor. We have always found the "Red Back" a clean and fair journal and a good advertising medium, as our continuance will testify.

Wishing you both many years of prosperity and happiness, we are, with kindest regards,

Yours truly,

ARTHUR PETER & CO.

[The above, after twenty-seven consecutive years' continuous patronage.—ED.]

BECKVILLE, TEXAS, June 5, 1912.

*F. E. Daniel, M. D., Austin, Texas.*

MY DEAR OLD FRIEND: I enclose check for $3.00, two of them to help you "crank up for the twenty-eighth-mile run." One for a copy of "The Strange Case of Dr. Bruno," which please send me.

May God bless you. Your old guard will never desert you.

Your friend,

M. D. STERRETT.

---

I WISH TO TAKE advantage of your offer if you are agreeable, and enclose express money order for $3.00.

I was thinking some of cancelling my subscription to the "Red Back," now that I live so far north, but I have postponed the evil day because I couldn't help feeling I should miss the warm breeze from the south that streams out from between the covers of the "Red Back" whenever I open it!

Yours very truly,

H. D. EATON, Shopiere, Wis.

Formerly of Chihuahua, Mexico, but now no longer in that ruined country, thank God!

---

THE TEXAS PHARMACEUTICAL ASSOCIATION held a three days' session in Austin, June 19-21, and transacted much important business. It went on record as opposed to the establishment by the State of a laboratory for the manufacture of sera for the prevention and treatment of meningitis, diphtheria, typhoid, etc. Meantime, State Health Officer Steiner announces through the press that such department has been established, and is now prepared to furnish the several antitoxins "at cost." The druggists, I believe, based their opposition on the great cost to the State. A meeting of the State Board of Pharmacy was held during the session, and some seventy druggists were examined and licensed to practice in Texas. Dr. H. C. Jackson, of Austin, was elected President; John Weeks, of Ballinger, W. D. Adams, of Forney, and H. Driess, of San Antonio, the Vice-Presidents; E. Eberle, of Dallas, Secretary-Treasurer. Next meeting, 1913, Galveston.

---

In performing external esophagotomy, the trachea is the guide for finding the esophagus. It is easy to remember that there is nothing but the esophagus between the trachea and vertebral column.—*American Journal of Surgery.*

